# Spontaneous passage of multiple ingested razor blades: A rare case report and review of the literature

**DOI:** 10.1097/MD.0000000000043857

**Published:** 2025-08-15

**Authors:** Liang Zhang, Shihui Li, Xiaoli Yang, Juan Zhou, Ying Li, Shumei Zheng, Shanhong Tang

**Affiliations:** a Department of Gastroenterology, General Hospital of Western Theater Command, Chengdu, Sichuan, China.

**Keywords:** blades, esophagogastroduodenoscopy, foreign body, perforation

## Abstract

**Rationale:**

Foreign body ingestion in children is a medical emergency, with severe cases posing life-threatening risks. Swallowing blades is an extremely rare and critical emergency requiring immediate medical intervention. The blades may cause perforation or bleeding in the gastrointestinal tract at any time, threatening the patient’s life. This case report aims to provide treatment process and experience for dealing with this condition.

**Patient concerns:**

A 14-year-old girl was hospitalized for swallowing 6 blades.

**Interventions and outcomes:**

Upon hospital admission, an emergency esophagogastroduodenoscopy was immediately performed. One blade was removed under emergency esophagogastroduodenoscopy, and the other 5 blades had entered the intestine and could not be removed endoscopically. After conservative medical treatment, the rest blades remarkably and naturally passed without surgical intervention. And the patient was discharged from the hospital without obvious discomfort.

**Lessons:**

Ingesting blades might lead to serious or fatal complications that require urgent surgical treatment. Strict medical management and timely assessment of the condition are very important for passage of ingested blades, which may avoid surgical procedures. This report provides valuable insights and guidance for clinicians in the management of patients with swallowing blades.

## 1. Introduction

Foreign body (FB) ingestion is common in children, which is an emergency for gastroenterologists and pediatricians. The common FB identified are coins, button batteries, magnets, jewelry, and sharp objects, such as needle and blades, are less commonly.^[1]^ Most foreign objects (about 60%) are in the stomach. 25% to 30% foreign bodies are in the esophagus and only 10% foreign bodies reach the small intestine.^[2]^ Most small and blunt foreign bodies can resolve naturally when they come with the feces after conservative treatment.^[3]^ Among them, button batteries represent a critical pediatric emergency distinct from other foreign bodies due to the chemicals they contain to cause corrosive damage to the digestive tract, such as mucosal burns, perforations, stricture and vocal cord paralysis.^[4]^ Therefore, if swallowed by mistake, one should immediately try to remove it. However, for long and sharp foreign bodies, it is easy to lead to mucosal damage, bleeding, perforation and other complications. When the foreign bodies reach the jejunum or ileum, and present clinical symptoms such as abdominal pain, nausea, and vomiting, the surgical rate is as high as 91.7%.^[5]^ Here we present a case of blades ingestion in a 14-year-old female patient which was managed conservatively with abdominal examination and abdominal computerized tomography (CT).

## 2. Case presentation

**Figure 1.**
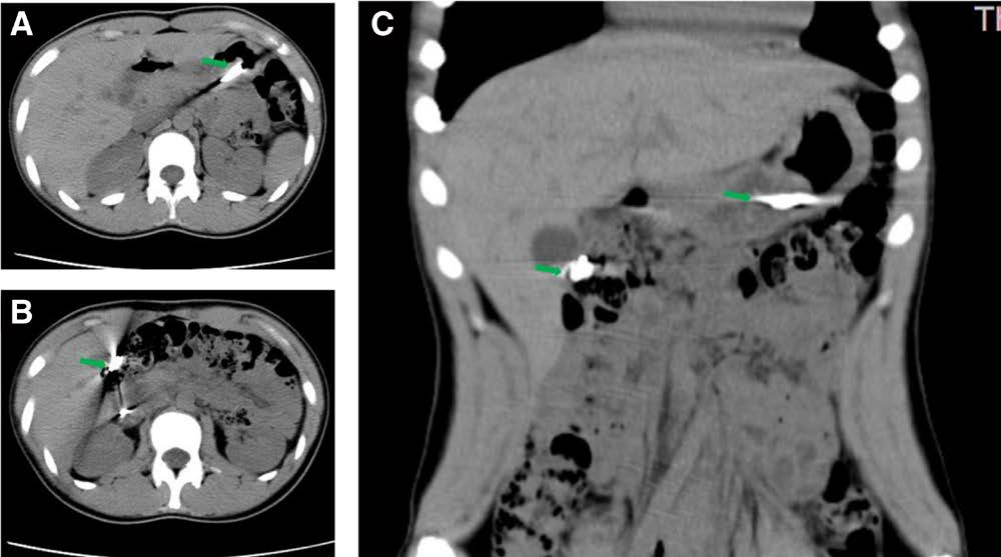
Abdominal CT showing dense shadows in the stomach and duodenum (A–C, green arrow). CT = computerized tomography.

**Figure 2.**
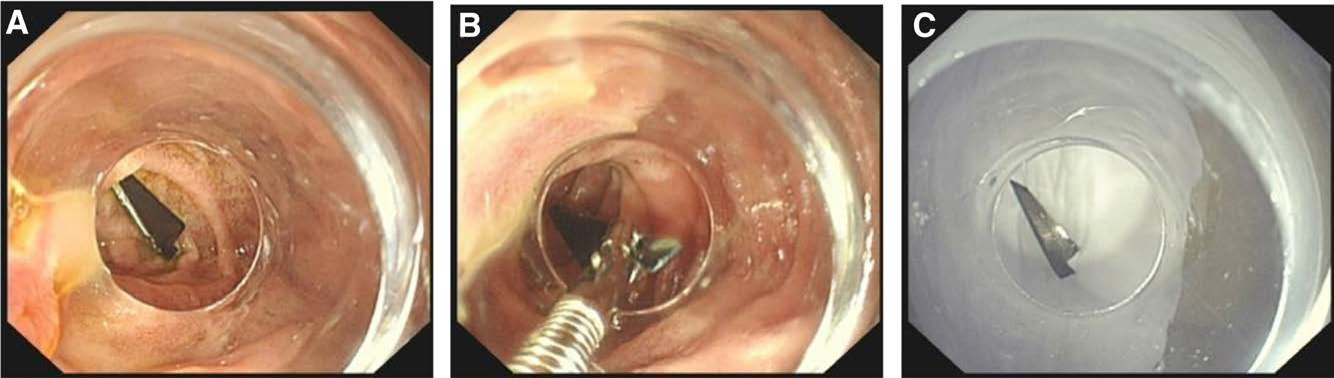
Gastroscopy showed that the blade was in the duodenum and removed with foreign forceps.

A 14-year-old girl with ingested blades for 13 hours was admitted to our hospital. The patient was in good health with no history of infection, trauma or surgery. The child swallowed 6 blades 13 hours before admission, and subsequently developed epigastric pain without nausea, vomiting, chest pain, chest tightness, and other symptoms. Then 4 hours later, she went to the local hospital, and abdominal radiograph suggested the possibility of intestinal FB. Immediately, she was sent to the emergency department of our hospital. An emergency abdominal CT examination revealed flaky dense shadows found in the stomach, duodenum and intestine, with obvious radiative artifacts around them (Fig. 1). The larger one was in the stomach, with a range of about 1.9 cm × 0.3 cm, indicating foreign bodies. No definite free gas was found in the abdominal cavity. The electrocardiogram showed sinus arrhythmia with a heart rate of 66 to 87 beats per minute. An emergency esophagogastroduodenoscopy was immediately performed. A 1.2 cm × 0.4 cm blade was found in descending duodenum and was removed by endoscope (Fig. 2). During the removal of blade, the patient vomited violently and cooperated poorly. After the blade was removed, the patient developed severe abdominal pain and obvious tenderness in the right upper abdomen. The possibility of perforation was suspected and the endoscopy procedure was suspended, then the patient was admitted to the department of gastroenterology.

**Figure 3.**
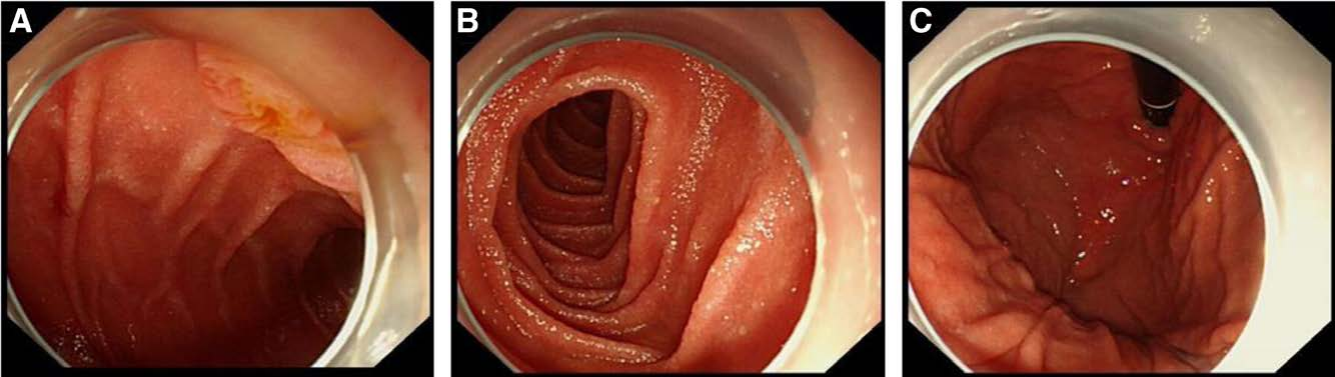
Gastroscopy showed scattered injury of mucous membrane in the greater curvature of the stomach with brown blood scab. No obvious abnormality was found in the duodenum.

**Figure 4.**
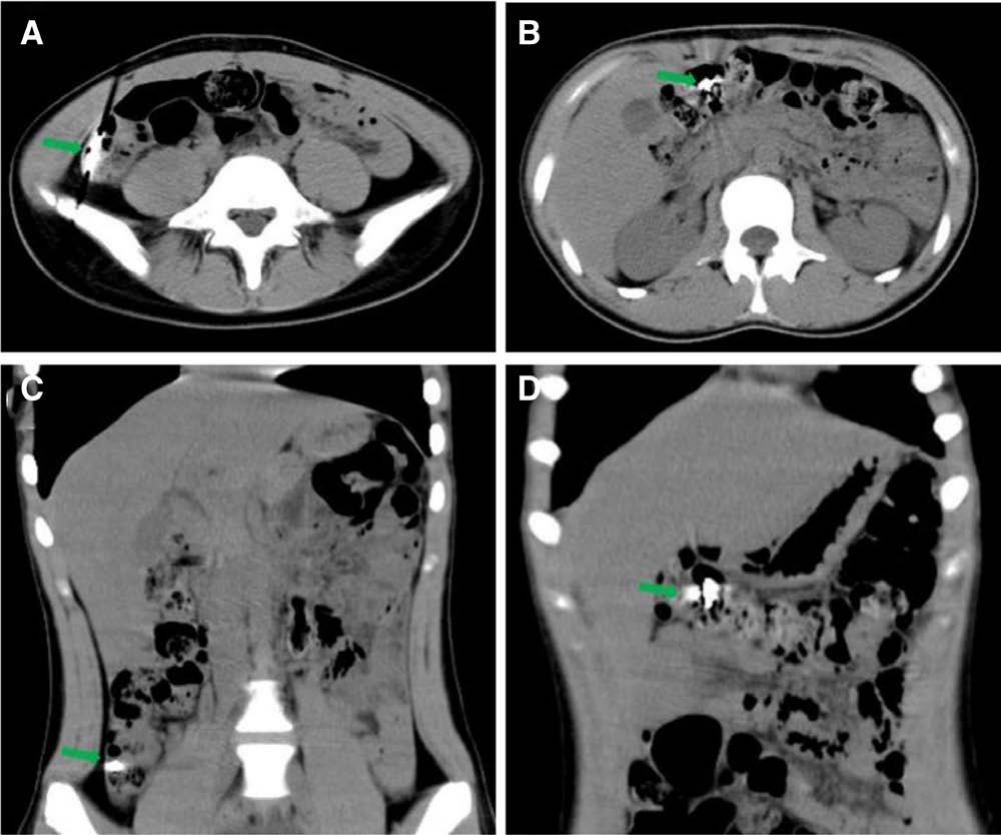
Abdominal CT showed 2 dense shadows in the ascending and transverse colon (A–D, green arrow). CT = computerized tomography.

**Figure 5.**
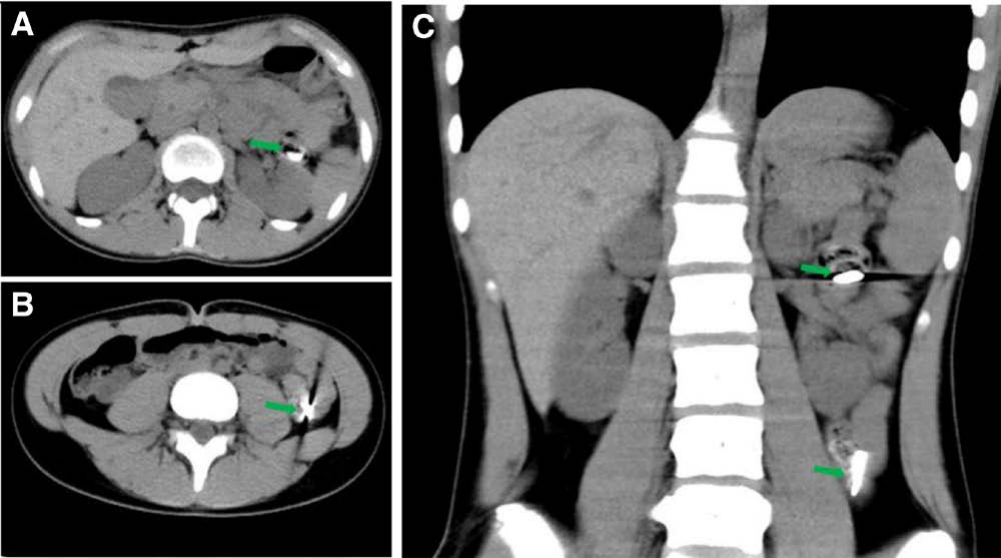
Abdominal CT showed 2 dense shadows in the transverse and descending colon (A–C). CT = computerized tomography.

**Figure 6.**
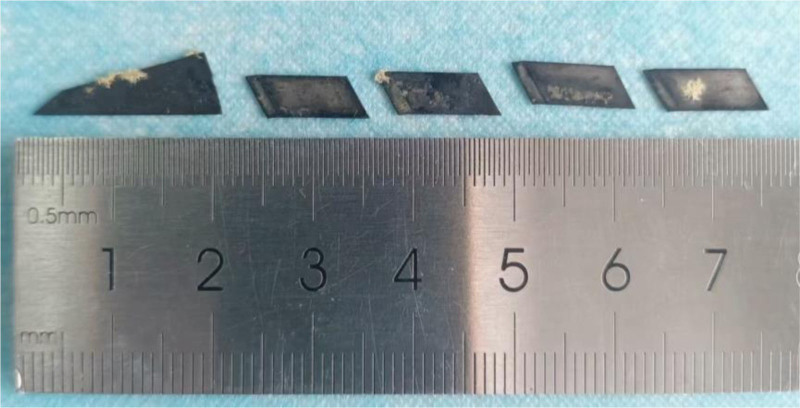
The blades the child swallowed, passed through her intestines.

On examination, the patient’s vital signs were within the normal range and physical examination showed no obvious abnormality. There was no generalized tenderness, guarding, or rigidity. Blood cell analysis revealed hemoglobin level, white blood cell and platelets counts of 139 g/L (normal range), 6.13 × 10^9^ L (normal range 3.5 × 10^9^–9.5 × 10^9^/L) and 239 × 10^9^ L (normal range), respectively. The hypersensitive C-reactive protein was under 0.50 mg/L (normal range 0–3 mg/L). Other biochemical indicators were within the normal range. After treatment with proton pump inhibitor and fluid rehydration, the patient’s general condition improved 6 hours after admission. Then, a repeated esophagogastroduodenoscopy under general anesthesia was performed and revealed scattered lesions of the mucosa of the greater curvature of the stomach but without obvious abnormalities in the duodenum (Fig. 3). Consider the remaining blades have entered the intestine, and the esophagogastroduodenoscopy cannot enter and remove them. We subsequently contact the gastrointestinal surgeon and reach a consensus that surgical treatment should be performed in time when there is perforation or gastrointestinal bleeding. The next day, the patient developed dull pain and discomfort in the right upper abdomen with intermittent colic. A second emergency abdominal CT scan (Fig. 4) showed 2 dense shadows in the ascending colon and transverse colon lumen, with obvious radial artifacts around them. After surgical consultation, there were no indications for surgery. In treatment, liquid paraffin was added to moisten the intestine. In the following 2 days, her condition was generally stable. A third abdominal CT plain scan showed (Fig. 5) 2 flake dense shadows in the transverse colon and descending colon lumen. Treatment with enema was performed and finally, 5 blades were found in the stool (Fig. 6). After observation and treatment, the patient had no obvious discomfort and was discharged at the request of the patient’s parents.

## 3. Discussion and conclusions

For foreign bodies in the digestive tract above the pylorus, most of them can be removed by endoscopy or in combination with various methods.^[6]^ However, for foreign bodies that have reached below the pylorus and cannot be removed by endoscopy, there are few reports. Wu et al retrospectively studied 16 cases of foreign bodies below the pylorus in children, among which the FB was a blade in only 1 case: the patient swallowed 3 blades, and after fasting and nutritional support treatment, the blades were excreted by itself.^[7]^ However, in our case, the patient swallowed 6 blades which was more than the former. Further, when the patient came to our hospital for gastroscopy, more than 10 hours had passed and the best time for endoscopic treatment has been missed. And only 1 blade was removed by endoscopy and the patient suffered severe pain during the process. The other sharp blades have reached below the pylorus and the risk of perforation or bleeding may occur at any time during the movement of blades along the intestine. Complications of perforation occur in 15% to 35% of the cases.^[2]^ The ileocecal valve and anus are the common sites for impaction or perforation characterized by acute angulations or physiological constriction. The patient’s condition is urgent without indication of surgical operation, which is very difficult and challenging for physicians. Throughout the course of treatment, fasting, rehydration and enema were used, while vital signs were closely monitored.

The best time for endoscopic removal of foreign bodies is not clear, and in principle, the earlier the better. Studies have shown that foreign bodies in the upper gastrointestinal tract should be removed within 24 hours, otherwise the risk of complications and the failure rate of endoscopic extraction will be significantly increased.^[8]^ It has been reported that retention of foreign bodies over 24 hours is an independent factor affecting the failure of endoscopic extraction.^[9]^ The European Society of Gastrointestinal Endoscopy recommends emergent therapeutic endoscopy for foreign bodies preferably be performed within 2 hours, but at the latest within 6 hours.^[10]^ Therefore, for sharp foreign bodies, endoscopy should be performed as soon as possible, and the time of endoscopic removal is best within 6 hours, especially for children.

Our case provides some valuable lessons worth learning for the removal of sharp foreign bodies in children. Firstly, gastroscopy under anesthesia can reduce patient pain and improve examination compliance, which is conducive to the removal of foreign bodies. In addition, intravenous anesthesia can also relax the smooth muscle of the esophagus, reduce the damage to the gastrointestinal mucosa when foreign bodies are removed. Secondly, it is important to note that imaging is essential to detect complications.^[11]^ Abdominal CT scan was performed every 12 hours after admission to observe the location of foreign bodies, which was helpful to evaluate whether there was bleeding and perforation, to timely adjust the treatment strategy. Finally, enema and oral administration of liquid paraffin were helpful to promote intestinal movement and removal of foreign bodies. The blade showed a downward performance along the intestine after the use of enema and liquid paraffin in the patient, suggesting that enema and liquid paraffin can assist the intestinal discharge of sharp foreign bodies. However, the frequency of use of enema and liquid paraffin has not been clearly defined at present, which needs further study.

As for the reason why the patient swallowed blades, we considered that the patient, in the teenage years, was facing great academic and social pressure, which lead to psychological disorders. Therefore, attention should be paid to the mental health problems of this population to prevent self-harm or impulsive behavior in time.

This case is rare and was successfully managed conservatively. Through reviewing treatment process of this case, close imaging evaluation and timely endoscopy are essential for the treatment of foreign bodies in gastrointestinal tract. We hope the case can provide some experience and thinking for clinicians.

## Acknowledgments

We would like to acknowledge our patient for her support.

## Author contributions

**Conceptualization:** Liang Zhang, Shihui Li, Shumei Zheng, Shanhong Tang.

**Data curation:** Liang Zhang, Shihui Li, Xiaoli Yang, Juan Zhou, Ying Li.

**Investigation:** Xiaoli Yang.

**Supervision:** Ying Li.

**Validation:** Shihui Li, Xiaoli Yang, Juan Zhou, Ying Li.

**Writing – original draft:** Liang Zhang.

**Writing – review & editing:** Shumei Zheng, Shanhong Tang.
